# The relationship between emotional intelligence, internet addiction, and psychological well-being among university nursing students

**DOI:** 10.1186/s12912-025-03957-2

**Published:** 2025-10-28

**Authors:** Nadia Kasem Alaswad, Shymaa Mohammed Sayed Hassan, Saleh Omar Abdullah, Hanan Azouz Abd Elhay Mohamed, Hanan Faried Maghawry

**Affiliations:** 1 Pediatric Nursing, Faculty of Nursing, Cairo University, Cairo, Egypt; 2 Psychiatric and Mental Health Nursing, Faculty of Nursing, Assuit University, Assuit, Egypt; 3 Nursing Department, Faculty of Nursing, Irbid National University, Irbid, Jordan; 4 Psychiatric and Mental Health Nursing, Faculty of Medicine & Health Sciences, Hodeidah University, Al Hudaydah, Yemen; 5 Pediatric Nursing, Faculty of Nursing, Assiut University, Assiut, Egypt

**Keywords:** Emotional intelligence, Internet addiction, Psychological well-being, Nursing students

## Abstract

**Background:**

Internet addiction (IA) is a behavioral condition characterized by impaired impulse control. Although it does not involve substances, it shares features with other behavioral addictions such as pathological gambling. This study aimed to examine the relationships among emotional intelligence (EI), internet addiction, and psychological well-being (PWB) among university nursing students.

**Methods:**

A descriptive correlational research design was employed with a stratified sample of 335 students from the Faculty of Nursing at Assiut University. Ethical approval was obtained from the Faculty’s Ethics Committee (IRB number: 1120250980). Data were collected using a demographic questionnaire, the Trait Meta-Mood Scale, the Internet Addiction Test, and Ryff’s Psychological Well-Being Scale.

**Results:**

Students with higher EI scores demonstrated higher mean ranks of IA (mean rank = 173.24) as well as higher mean ranks of PWB (mean rank = 183.81). Correlation analyses revealed weak but statistically significant associations among the three variables (*r* ≈ 0.15–0.29). Specifically, EI was positively correlated with both PWB and IA, while IA was negatively correlated with PWB.

**Conclusion:**

Nursing students demonstrated relatively high EI, moderate levels of IA, and moderate to high well-being range based on the scale cutoffs. Although the associations between EI, IA, and PWB were statistically significant, the effect sizes were small, limiting their practical significance. The negative relationship between IA and PWB is consistent with prior literature, underscoring the potential adverse effects of excessive internet use on students’ mental health. While integrating EI training into nursing education may be beneficial, the findings should be interpreted cautiously due to the weak correlations observed.

**Clinical trial number:**

Not applicable.

**Supplementary Information:**

The online version contains supplementary material available at 10.1186/s12912-025-03957-2.

## Background

The American Psychiatric Association’s *Diagnostic and Statistical Manual of Mental Disorders*, Fifth Edition (DSM-5), has identified Internet Addiction (IA) as a proposed psychological disorder for inclusion [1]. In the modern era, IA was categorized under impulse control disorders, bearing similarities to pathological gambling but without involving substance abuse. This condition poses a significant threat to mental health and may negatively impact emotional intelligence (EI) and psychological well-being (PWB) [2].

Excessive engagement with the internet, often accompanied by emotional disturbances, is increasingly recognized as a behavioral concern. This pattern of behavior, commonly referred to as Internet Addiction (IA), is characterized by an inability to control the urge to go online, leading to compulsive use and obsessive thoughts about internet-related activities. Individuals affected by IA often struggle to manage their online habits, resulting in significant impacts on their mental and emotional well-being [3, 4].

Technological addiction including IA is considered a psychological and social disorder in which emotional dysregulation plays a critical role in the development of addictive behaviors [5, 6]. In this regard, Far et al. and Berte et al. [7, 8] emphasized that EI refers to the capacity to recognize, understand, and manage one’s own emotions as well as those of others, particularly in the context of social interactions. EI helps clarify the role of emotions in human functioning and supports the development of self-awareness and emotional self-regulation.

According to Maghawry et al. [9], EI significantly influences how individuals navigate social environments. Unlike cognitive intelligence, EI encompasses the ability to interpret and harness emotions, manage mood fluctuations, control impulses, and exhibit strong interpersonal skills. These competencies are essential for effectively managing both everyday social demands and personal challenges. Similarly, Naeem et al. [10] define PWB as the capacity to actively engage across multiple dimensions of health, including physical, intellectual, emotional, spiritual, social, and environmental aspects.

With growing global concern over excessive internet use among students, it is vital to acknowledge both the advantages and challenges posed by digital connectivity [11]. While the rise of the internet has revolutionized information exchange and spurred technological advancement, it has also contributed to maladaptive usage patterns, including addictive behaviors among university students. In this context, EI plays a pivotal role by enhancing emotional and social competencies that serve as protective factors against internet-related addictions [12, 13].

Research has shown that EI fully mediates the relationship between IA and mental health, suggesting that enhancing EI may reduce the adverse effects of IA on psychological well-being [14]. El-Ashry et al. [15], in their study titled *Hooked on Internet: Examining the Co-occurrence of Nomophobia and Impulsive Sensation Seeking Among Nursing Students*, explored how traits such as impulsivity and sensation-seeking are often linked to lower EI associated with higher rates of nomophobia and IA. These traits were also correlated with poor coping strategies and elevated psychological distress, indicating a harmful impact on PWB.

Similarly, Mohammed et al. [16], in their study *Internet Addiction Among Middle Eastern Students Significantly Impacts Mental Health*, reported that excessive internet use among university students, including those in nursing programs, was strongly associated with increased levels of anxiety, depression, and decreased life satisfaction. Collectively, these findings suggest that insufficient EI may heighten the vulnerability of nursing students to technology-related addictions, including IA, thereby compromising their psychological health. Enhancing EI through targeted mental health and educational interventions may therefore be an essential strategy to foster resilience, mitigate the negative consequences of internet overuse, and safeguard the well-being of nursing students.

The theoretical basis for examining the interrelationship between EI, IA, and PWB, the present study adopts Gross’s (1998) Emotional Regulation Theory. Savarimuthu et al., [17] mentioned that emotional regulation theory posits that individuals use various cognitive and behavioral strategies to influence their emotional experiences, expressions, and responses. Emotional regulation is central to EI, which involves recognizing, managing, and utilizing emotions effectively. Also, Kämpf et al., [18] stated that poor emotional regulation may contribute to maladaptive coping behaviors such as excessive internet use, leading to decreased PWB.

**Fig. 1:**
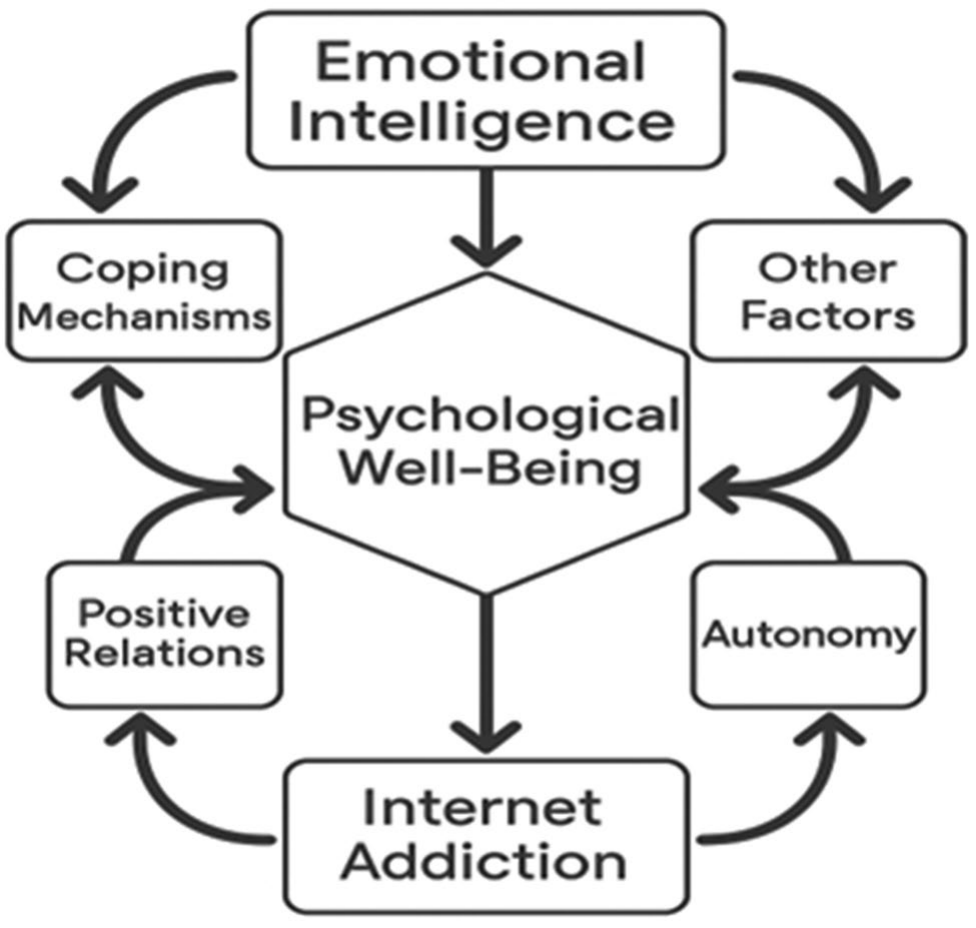
Illustrate the relationship among study’s variables. Developed by research investigators

By applying this framework, the study aims to understand if EI serves as a protective factor, potentially buffering the negative effects of IA on students’ psychological health. Thus, Emotional Regulation Theory provides a meaningful lens for interpreting the mechanisms linking the study’s main constructs Fig. 1. Defense mechanisms, such as denial, repression, and projection, may further contribute to this dynamic by obscuring emotional awareness and encouraging avoidance behaviors. For instance, a student experiencing social anxiety may unconsciously use internet use as a way to suppress interpersonal discomfort, leading to compulsive online behavior. Additionally, factors such as social support, academic stress, personality traits (e.g., neuroticism or resilience), sleep patterns, and coping style can moderate these relationships [19].

Students with strong social networks and problem-focused coping strategies are more likely to regulate emotions constructively, reducing reliance on the internet as a coping tool. In contrast, those with poor support and emotion-focused or avoidant coping styles are at higher risk of developing internet addiction and experiencing diminished well-being [20, 21]. In sum, Emotional Regulation Theory provides a valuable framework for understanding how emotional intelligence can buffer the negative effects of internet addiction and enhance PWB, especially when supported by adaptive coping mechanisms and healthy environmental factors [22].

The theory emphasizes two broad categories of regulation strategies: antecedent-focused strategies (such as cognitive reappraisal) and response-focused strategies (such as suppression). Individuals with higher EI are expected to engage more frequently in adaptive strategies, which in turn promote psychological well-being [17]. However, research has also indicated that the same emotional awareness and sensitivity associated with EI may increase vulnerability to maladaptive coping when stressors are high and adaptive strategies are either unavailable or ineffective [21].

In this context, internet use can function as a form of emotion regulation, sometimes adaptive (e.g., distraction, social connection), but also potentially maladaptive when it develops into excessive or compulsive use [20]. Integrating Gross’s theory, we propose that the relationship between EI and internet addiction (IA) is not strictly linear: while EI is generally protective, in certain contexts it may predispose students to use the internet as a maladaptive coping mechanism, thereby explaining counterintuitive associations between high EI and higher IA.

### Significance

The growing number of internet users has led to a rise in IA, especially among specific populations such as university nursing students [23, 24]. Moreover, the importance of growing the work on EI in university students of health-related professions including nursing hence their activities is directed toward humans, facing real situations with human emotions so, the capability of those students to manage their own emotions properly enable them to understand and develop proper communication channels targeted in care provided leads to high quality performance and satisfaction for both patient and nurses [25, 26].

Understanding the relationship between EI, IA, and PWB among university nursing students is critical due to the high emotional and cognitive demands of the nursing profession. This research adds to existing knowledge by examining the potential of EI to mitigate adverse psychological outcomes particularly in academic settings. By identifying the interconnections among these variables, the research can provide insight into the development of specific interventions aimed at enhancing emotional resilience, reducing internet-related behavioral risks, and promoting overall mental health in future healthcare professionals.

### Aim of the study

This research aims to explore the relationship between emotional intelligence, internet addiction and psychological well-being among university nursing students.

## Methods

### Research questions

Q1: What is the extent of emotional intelligence, internet addiction and psychological well-being among university nursing students?

Q2: Is there a correlation between emotional intelligence, internet addiction and psychological well-being among university nursing students?

### Study design

A descriptive correlational research design used in this study.

### Study setting

This study conducted at Assiut University’s Faculty of Nursing.

### Subjects

The study subjects consisted of male and female nursing students from the first to fourth academic years during the academic year 2024/2025.

### Sample size figure 2

A Stratified sample used for this study for the selected group of students from the first to the four academic years during the academic year 2024/2025; The total number of students enrolled in the Faculty of Nursing during the academic year 2024–2025 was 2,473 students, distributed according to their academic year as follows: 1st year (*n* = 573), 2nd year (*n* = 560), 3rd year (*n* = 660), and 4th year (*n* = 680).

Data were entered and analyzed using Epi Info version 3.0, developed by *Disease Control and Prevention* (CDC), which allowed for custom form creation, data entry validation, and basic statistical and epidemiological analyses. The required sample size estimated is based on the following parameters: population size = 2,473 students and a confidence level of 95%.

Valid data estimated: Epi-info program version 3.0 was (*n* = 335).

**Fig. 2:**
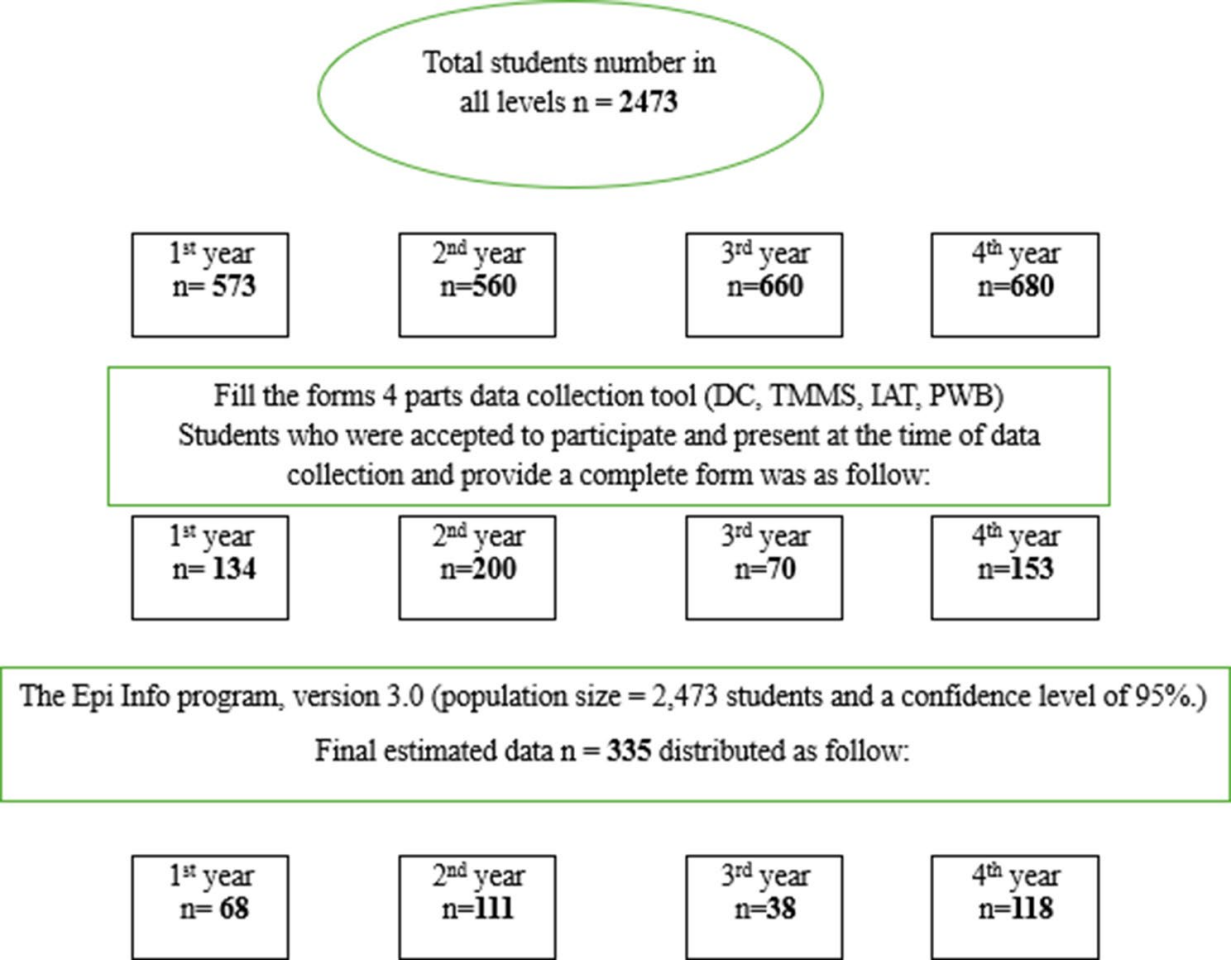
Sample size estimation process

### Data collection tools

Consisted of four parts:

#### Tool (1): Demographic characteristics of the students (DC)

This section was developed by the researcher in Arabic and included variables such as age, sex, academic year, and place of residence. A jury of five experts in psychiatric and mental health nursing and pediatric nursing was asked to assess the importance and relevance of each item on the scales. They also reviewed the tools for clarity, relevance, comprehensiveness, understandability, and applicability. Revisions were made based on their suggestions and the necessary modifications.

#### Tool (2): Trait Meta-Mood scale (TMMS)

The translated version (English and Arabic) of the study’ used scales available in supplementary files.

Salovey et al. [27] conceptualized emotional intelligence as individuals’ self-perceptions of their emotional abilities, referring to this construct as “trait meta-mood.” This framework assesses how individuals believe they perceive, understand, and regulate their emotions. A 24-item Spanish-adapted scale, originally developed by Del-Carmen and Prado [28], was used to measure this construct. The instrument evaluates the extent to which individuals pay attention to their emotions, interpret emotional experiences, recognize others’ emotional expressions, and regulate their mood to fit social environments.

Responses are recorded on a 5-point Likert scale ranging from 1 (never) to 5 (always).

The scale includes three subcomponents: Emotional attention (items 1–8), Clarity of feelings (items 9–16) and Mood repair (items 17–24, excluding item 22). Total emotional intelligence scores range from 24 to 120, categorized as follows: Low: 24–55, Moderate: 56–87 and High: 88–120. The subscales demonstrated strong internal consistency in previous studies, with Cronbach’s alpha values ranging from 0.80 to 0.87 [22].

In the current study Although the original tool was developed in Spanish, the Arabic version used in this study was translated from its validated English version which translated using the standard forward–backward translation method. The translation process of English version followed a forward backward method to ensure conceptual and linguistic equivalence. Content validity was evaluated by a jury of five academic experts, two specialized in pediatric nursing and three in psychiatric nursing who reviewed the items for clarity, cultural relevance, and appropriateness. Revisions were made based on their feedback. The internal consistency (Table 1) of the Arabic version was tested using Cronbach’s alpha, demonstrating an acceptable level of reliability was 0.80.

#### Tool (3): Internet addiction test (IAT)

The Internet Addiction Test (IAT), originally developed by Young [29], is a widely used instrument to assess the degree of internet dependency. It consists of 20 items, each rated on a 6-point Likert scale from 0 (never) to 5 (always), based on how often participants engage in specific online behaviors (e.g., “How often do you stay online longer than intended?”). Total scores range from 0 to 100 and are interpreted as follows, according to Widyanto and McMurran [30]: 0–19: No signs of addiction, 20–39: Mild addiction/normal internet use, 40–69: Moderate addiction and 70–100: Severe addiction. The original tool was translated from English to Arabic using a standard forward-backward translation method to ensure linguistic and conceptual equivalence. The translated version was then reviewed for content validity by a jury of five academic experts, including two professors specializing in pediatric nursing and three in psychiatric nursing. Their feedback was incorporated to ensure cultural relevance and clarity of the items. To assess reliability, the internal consistency of the Arabic version was measured using Cronbach’s alpha test, which demonstrated an acceptable level of reliability (0.91) – Table (1) for use in the current study.

#### Tool (4): Ryff’s Psychological well-being scale (PWB)

The Psychological Well-Being Scale (PWB), initially developed by Ryff and Keyes [31] and refined by Ryff in 2013, is designed to assess six core dimensions of psychological well-being: Autonomy, Self-acceptance, Environmental mastery, Purpose in life, Personal growth, and Positive relations with others. The short version used in this study includes 18 items, with three items representing each dimension. Participants respond using a 6-point Likert scale ranging from 1 (“strongly disagree”) to 6 (“strongly agree”). The total score ranges from 18 to 108 and is categorized as follows: Low well-being: 18–48, Moderate well-being: 49–78, and High well-being: 79–108. The scale has demonstrated acceptable internal consistency in previous studies, with a Cronbach’s alpha of 0.89 and validation through content analysis [32].

The original tool was translated from English to Arabic using a standard forward-backward translation method to ensure linguistic and conceptual equivalence. The translated version was then reviewed for content validity by a jury of five academic experts, including two professors specializing in pediatric nursing and three in psychiatric nursing. Their feedback was incorporated to ensure cultural relevance and clarity of the items. To assess reliability, the internal consistency of the Arabic version was measured using Cronbach’s alpha test, which demonstrated an acceptable level of reliability 0.80, Table 1 for use in the current study.

### Ethical considerations

The study protocol was approved by the Ethical and Scientific Committee, with ethical approval granted under the code: 1,120,250,980. After explaining the aim and nature of the study, students who agreed to participate voluntarily signed an informed consent form. Participants were informed of their right to refuse or withdraw from the study at any time without any consequences. Prior to data collection, the research investigators emphasized that participants’ privacy would be fully respected and that all collected information would remain strictly confidential. Data were then collected from the defined study sample by the researcher. This study adhered to the principles outlined in the Declaration of Helsinki: (http://www.wma.net/en/30publications/10policies/b3/index.html) [33] regarding research conducted on humans and human data.

### Data security

All data collected during the study were handled with strict confidentiality and protected using standardized data security protocols. Electronic data were stored on password-protected computers and encrypted storage systems accessible only to the research team. Hard copy forms were kept in locked cabinets in secure locations. Personal identifiers were coded to ensure anonymity, and data were used solely for research purposes. The research team adhered to institutional and national ethical guidelines regarding data protection and privacy, including compliance with Ethical and Scientific Committee standards, e.g., the General Data Protection Regulation (GDPR), and institutional data protection policies]. No data were shared with third parties, and all participants were informed about the measures taken to secure their information prior to providing consent.

### Administrative phase

Official permission to conduct the study and collect data was obtained from the Dean of the Faculty of Nursing, Assiut University. This approval was communicated to the faculty members responsible for the relevant sections through both verbal and written official correspondence.

### The pilot study

A pilot study was conducted on 33 students from the total sample to test and evaluate the clarity, feasibility, and applicability of the research instruments, as well as to estimate the time required for data collection. The tools remained unchanged, as they were found to be clear and easily understood by the students. Therefore, the participants from the pilot study were included in the main study sample.

**Table 1:** Alpha cronbach’s coefficient for the scales included in the study to test reliability of tools and the result in the following table

Reliability Statistics CI 95%		
The scale	Cronbach’s Alpha	*N* of Items
TMMS	0.86	24
IAT	0.91	20
PWB	0.80	18

CI: Confidence intervale

Developed by research investigators

### Procedure

#### Implementation phase

Data collection began in the second week of February and concluded in the first week of April 2025. The research investigators coordinated with the teaching staff responsible for the relevant student groups to schedule the fieldwork. The preferred times for data collection either at the beginning or end of the class sessions were determined in consultation with the instructors. Data collection was carried out in alignment with both the researchers’ availability and the students’ academic schedules.

The research investigators introduced themselves to the students, explained the purpose and significance of the study, and emphasized its potential contribution to improving the educational process at their college. Assurance of full confidentiality was provided. Students were invited to participate voluntarily and were asked to give their informed consent.

The main components of the research instruments were explained, and detailed instructions were given on how to complete the questionnaire. Hard copy forms were distributed to students who agreed to participate. On average, it took each participant 15 to 25 min to complete the questionnaire, depending on individual response times. The process was supervised by both the research investigators and the teaching staff present during the data collection sessions.

### Statistical analysis

The collected data were tabulated and analyzed using the Statistical Package for the Social Sciences (SPSS), version 20. Categorical variables were presented as frequencies and percentages, while continuous variables were described using means and standard deviations. Descriptive statistics were used to analyze the demographic characteristics of the students. To examine relationships between study variables, nonparametric tests (Kruskal-Wallis) were employed, effect sizes for Kruskal–Wallis tests were estimated using epsilon squared (ε²). Bootstrapping with 1,000 replications was applied to generate 95% confidence intervals as appropriate. When Kruskal–Wallis tests were significant, post-hoc pairwise comparisons were conducted using Dunn’s test with Bonferroni correction for multiple testing. Effect sizes for pairwise contrasts were calculated using rank-biserial correlation (r), the Bonferroni correction was chosen because it provides a conservative adjustment, thereby minimizing the risk of Type I error in the multiple pairwise comparisons. The Spearman correlation was used to assess the strength and direction of association between continuous or ordinal variables, such as the relationships between emotional intelligence, internet addiction, and psychological well-being. Correlation coefficients were used to assess associations between continuous variables. A p-value of less than 0.05 was considered statistically significant.

## Results

**Table 2:** Demographic characteristics of the students (*N* = 335)

	*N*	Percent (%)
**Sex**		
Male	156	46.6
Female	179	53.4
**Academic year**		
1st year	68	20.3
2nd year	111	33.1
3rd year	38	11.3
4th year	118	35.2
**Residence**		
Rural	199	59.4
Urban	136	40.6

Table 2 shows that the majority of the students were female (53.4%), with a substantial portion enrolled in the 2nd (33.1%) and 4th (35.2%) academic years. Additionally, about two-thirds of the students were from rural areas (59.4%).

**Table 3:** Correlation between the study variables and nursing students’ gender

	Sex	Mean Rank	*p*-value	r
EI^1^	Male	157.97	0.77	0.01
	Female	176.74		
IA^2^	Male	163.17	0.39	0.002
	Female	172.21		
PWB^3^	Male	161.37	0.24	0.004
	Female	173.78		

Effect sizes (ε²) were calculated for Kruskal–Wallis tests (SPSS), with 95% confidence intervals obtained via bootstrapping (1,000 resamples)

^1^Emotional intelligence

^2^Internet addiction

^3^Psychological well being

Table 3 indicates that while females showed higher mean ranks across all study variables, the gender differences were not statistically significant (*p* > 0.05) with small effect size (ε*²= 0.01*,*0.002*,*0.004)*.

**Table 4:** Kruskal–Wallis and post hoc pairwise comparisons of EI, IA, and PWB across academic years (*N* = 335)

Variable	Academic Year (Mean Rank)	Omnibus *p*	Compact Letter	Significant Pairwise Contrasts	Adj. *p* (Bonferroni)	Effect size (*r*)	Interpretation
EI	1st = 164.93, 2nd = 174.15, 3rd = 173.92, 4th = 162.08	0.810	–	None	–	–	Not significant
IA	1st = 137.06, 2nd = 142.25, 3rd = 204.90, 4th = 198.20	< 0.001*	a = (1st, 2nd); b = (3rd, 4th)	1st–4th < 0.001, 1st–3rd = 0.003, 2nd–4th < 0.001, 2nd–3rd = 0.003,	0.001–0.003	–0.19 to –0.24	Small–Medium
PWB	1st = 171.10, 2nd = 191.86, 3rd = 181.03, 4th = 139.58	< 0.001*	a = (2nd); b = (1st, 3rd, 4th)	2nd–4th < 0.001	< 0.001	0.22	Small–Medium

Compact letters indicate groups that are not significantly different from each other. Pairwise contrasts used Bonferroni-adjusted p-values. Effect sizes are reported as rank-biserial r. Interpretation follows Cohen’s (1988) guidelines: small (0.10), medium (0.30), large (0.50)

As shown in Table 4, IA and PWB significantly varied across academic levels (*p* < 0.001). Third-year students had the highest IA mean rank, whereas second-year students reported the highest PWB mean rank. For IA, post hoc pairwise comparisons with Bonferroni correction indicated that both first- and second-year students scored significantly lower than third- and fourth-year students. Effect sizes ranged from small (*r* = 0.19) to small–medium (*r* = 0.24).

No significant differences were found between first- and second-year students or between third- and fourth-year students. A compact letter display (see column 4 “Compact Letter” in Table 4) shows that first- and second-year students clustered in one group (a), while third- and fourth-year students clustered in another (b).For PWB, post hoc tests revealed that second-year students scored significantly higher than fourth-year students (*p* < 0.001, *r* = 0.22, small–medium). No other pairwise differences reached significance. The compact letter display indicated that second-year students formed a distinct group (a), whereas first-, third- and fourth-year students clustered together (b).

**Fig. 3:**
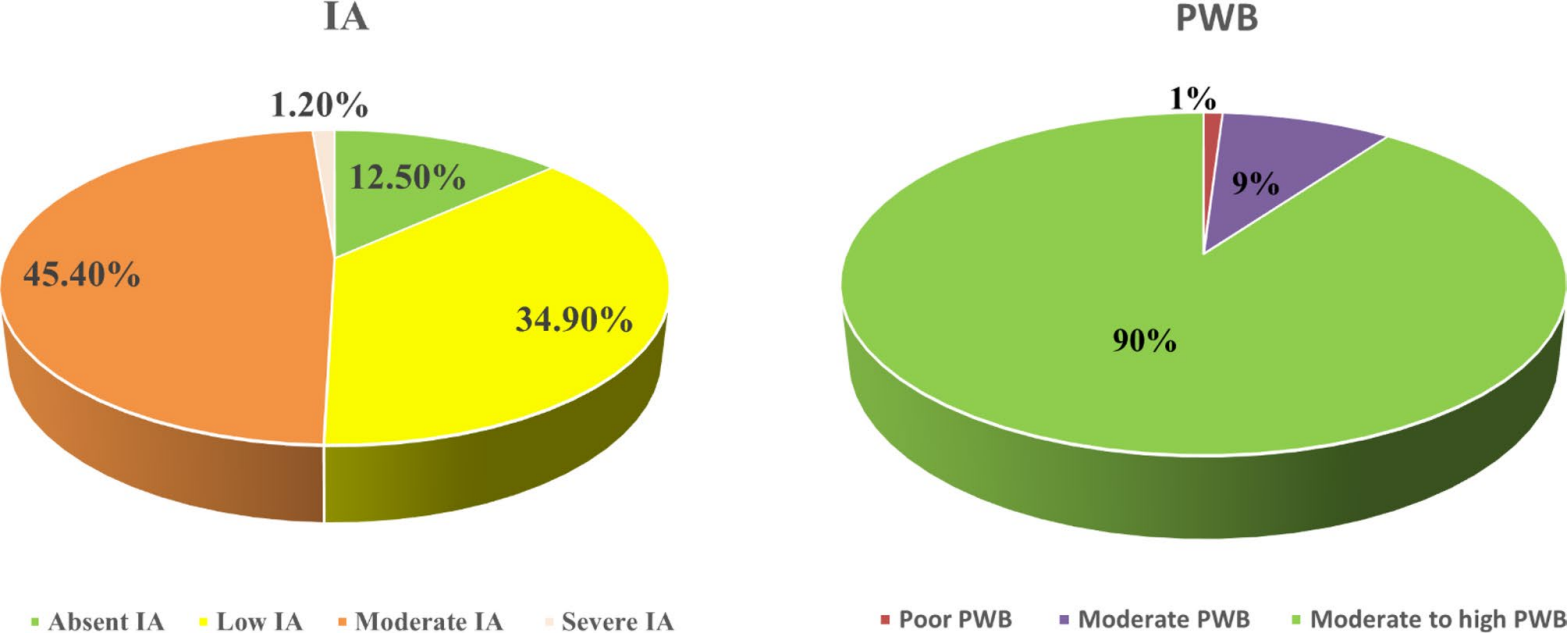
Descriptive data of levels of IA and PWB

According to the Fig. 3 and 45.4% of students demonstrated a moderate level of IA, while more than one-third had a low level of IA. Additionally, 90% of the students exhibited moderate to high PWB range based on the scale cutoffs.

**Fig. 4:**
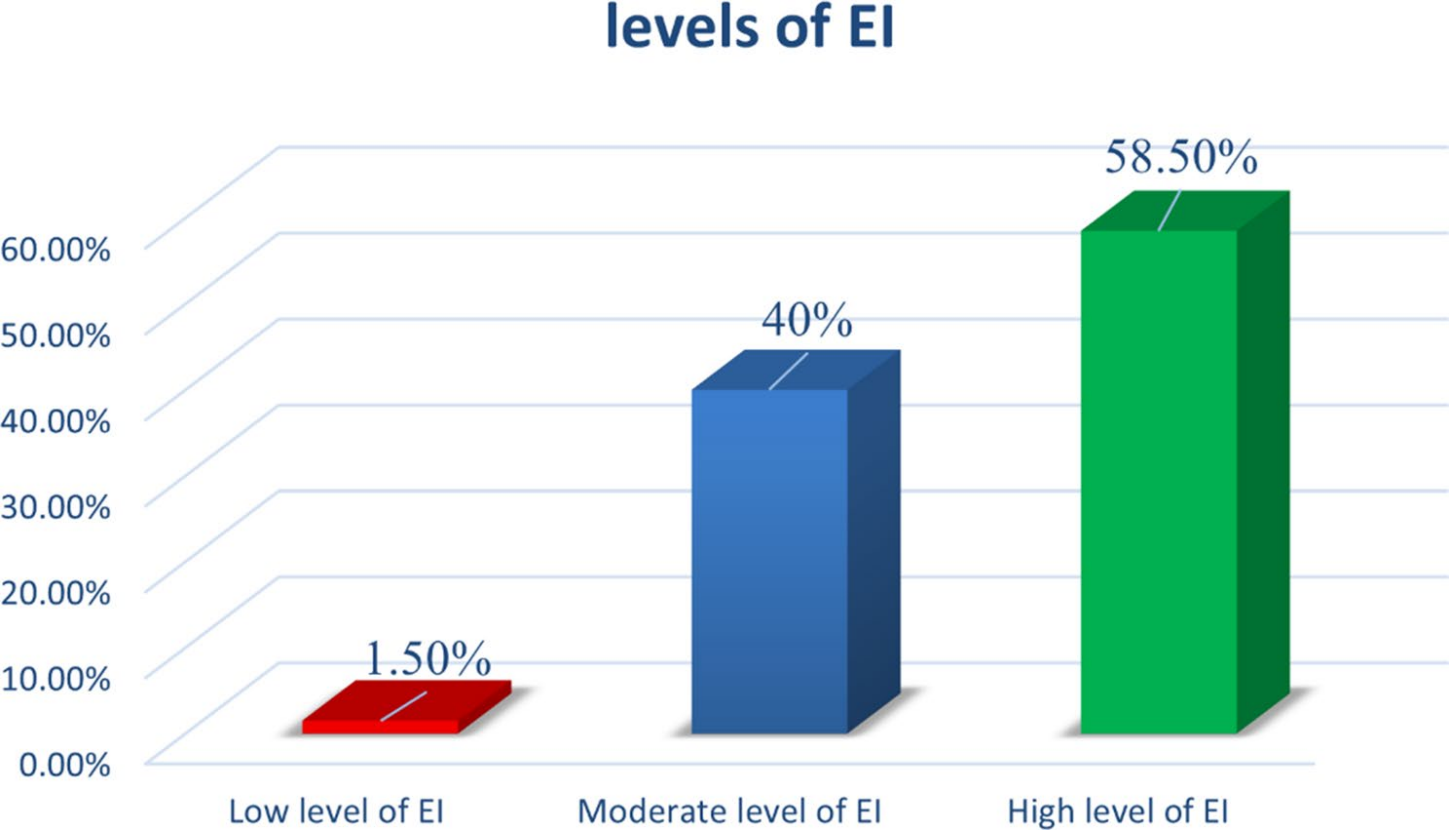
The students’ levels of (EI)

The Fig. 4 illustrates that the highest percentage of the participants had a high level of EI (58.5%), followed by moderate and low levels at 40% and 1.5%, respectively.

**Table 5:** Correlation between levels of EI and IA among nursing students

EI	IA	*p*-value	*Eta* ^*2*^
	Mean rank		
Low level of EI	12.00	0.001*	**0.035**
Moderate level of EI	166.15		
High level of EI	173.24		

Kruskal–Wallis test in SPSS *The statistical significance level was set at *p* < 0.05

As shown in Table 5 high-EI students had the highest IA scores, though the effect size was small.

**Table 6:** Correlation between levels of EI and PWB among nursing students

EI	PWB	p-value	*Eta* ^*2*^
	Mean Rank		
Low level of EI	169.90	0.002*	**0.033**
Moderate level of EI	144.80		
High level of EI	183.81		

Kruskal–Wallis test in SPSS *The statistical significance level was set at *p* < 0.05

As shown in Table 6, students with high emotional intelligence had the highest PWB scores, with a statistically significant difference, though the effect size was small.

**Table 7:** Inter- correlation between the study variables

	EI		IA		PWB	
	*r*	*p*	*r*	*p*	*r*	*p*
EI	1		0.294^**^	0.000*	0.151^**^	0.006*
IA			1		− 0.218-^**^	0.000*
PWB					1	

Spearman’s rho test in SPSS *The statistical significance level was set at *p* < 0.05

** small effect size

As shown in Table 7, emotional intelligence was positively correlated with both internet addiction (*r* = 0.294, *p* < 0.001) and psychological well-being (*r* = 0.151, *p* = 0.006), while internet addiction was negatively correlated with psychological well-being (*r* = -0.218, *p* < 0.001). All correlations were statistically significant but with small effect sizes.

## Discussion

This study aimed to investigate the connection between EI, IA, and PWB in nursing students. While the internet offers valuable benefits such as improved communication and access to knowledge, its diverse functions can also contribute to problematic usage patterns and difficulties in setting healthy boundaries.

Regarding demographic characteristics of nursing students, the greater portion of them were females, and about a substantial majority of them were in the 2nd and 4th academic years. Also, about two thirds of students were rural residents. these results agreed about gender with El-Gazar et al., [34] who found that larger portion of the studied subjects were females, however, disagree in residence and academic year as about two third were from urban and nearly a quarter were in the 4th academic year.

The current study revealed no statistically significant differences between male and female nursing students across the study variables. However, females consistently demonstrated higher mean ranks across all measured variables. These findings partially align with those of Lin et al. [35] and Rigelský et al. [36], who reported that female students are more likely to engage in online streaming and social networking, whereas male students typically exhibit higher levels of moderate to severe internet addiction, particularly through activities such as online gaming. Despite this, Ilk, & Güler [37] reported that the differences in internet addiction levels based on gender may not be as pronounced in certain contexts, indicating a need for further research to explore these dynamics comprehensively.

The absence of significant gender differences in the present study may reflect a growing trend toward gender parity in psychological and behavioral constructs among nursing students. This could be attributed to increasingly homogenized educational environments and the similar academic demands and pressures experienced by both male and female students.

Gender-based differences in emotional processing and internet use have been widely documented. For instance, prior studies. suggest that females often score higher in emotional intelligence, particularly in emotional awareness and empathy, while males may be more prone to externalizing behaviors, including maladaptive internet use [38, 39]. Despite these findings, some studies, such as one involving adolescent, reported no significant gender differences in emotional intelligence, suggesting that the gap may vary by age or context [40]. This highlights the complexity of emotional intelligence as a construct influenced by various factors beyond gender alone.

The present study demonstrated statistically significant differences in IA and PWB across academic levels among nursing students, Specifically, third-year students exhibited the highest mean rank for IA, closely followed by fourth-year students. These findings contradict Moitra & Chaudhari [41], who found a notable prevalence among first- and second-year students. The current study findings may reflect increasing academic and clinical demands during the later years of nursing education, which could lead nursing students to use the internet more frequently either for academic escape, stress relief, or maladaptive coping strategies.

In contrast, PWB showed a different pattern, with second-year students achieving the highest mean rank, followed by third-year students. These findings agree with Okasha et al., [42] who stated that nursing students encounter rigorous curricula, long teaching hours, competitive environment and frequent assessments, which contribute to chronic stress which affect students’ PWB.

This suggests that PWB tends to decline as students’ progress through their academic journey, possibly due to the cumulative stress of clinical exposure, academic performance pressures, and reduced personal time. First-year students may not have yet fully engaged with the clinical components, while final-year students may face increased anxiety regarding graduation and future employment.

Regarding the level of IA, the study found that approximately half of the students exhibited a moderate level of IA, while more than one-third demonstrated a low level. In addition, the majority of students showed moderate levels of PWB. These findings are consistent with those of Lebni et al. [43], who also reported a moderate level of IA among their participants. Similarly, Leodoro and Labrague [44] observed moderate levels of maladaptive internet use among nursing students. However, these results contrast with the findings of Díaz-Aguado et al. [45] and Seki et al. [46], who reported a high prevalence of IA among student populations in their respective studies.

The increasing incorporation of the internet into everyday routines highlights the need to closely examine internet-related addiction, especially among nursing students, given its possible effects on their academic performance and professional growth. Such addiction is typically marked by a compulsive need to stay online, which can lead to harmful outcomes in multiple aspects of life. It may weaken EI, increase psychological strain, lower academic productivity, and obstruct both learning and future career advancement.

This may explain why the students had moderate to high well-being range with moderate and low levels of IA. The study highlighted that more than half had a high level of EI, and two fifths had moderate level of EI. This finding was supported by El-Gazar et al., [34] who found an average EI score on a 5-point scale, suggesting that participants demonstrated a moderate or neutral level of EI.

This study found that students with high EI had the highest mean rank for IA, with a statistically significant correlation with weak association between the two variables. Additionally, students with high EI levels also showed the highest mean ranks in PWB, which was statistically significantly correlated with weak association as well. This result might support the claim of Fernández et al., [24] in their study about Internet dependency among university nursing students their study findings on nursing students clarified that there was a weak relationship between EI and IA.

These findings may be explained by the growing integration of the internet in the nursing field, including advancements like artificial intelligence and telehealth that are reshaping patient care. The widespread implementation of digital tools such as electronic health records and virtual healthcare services is generally viewed as a means to improve both efficiency and patient outcomes in clinical practice. Those who wish to remain up-to-date and progress in their careers ought to enhance their comprehension of the internet’s effects on nursing and the anticipated changes in the profession due to technological use; these challenges act as burden to nursing students to catch up with the new in health technologies.

Furthermore, the observed positive but weak correlation between EI and IA may initially appear counterintuitive; however, when interpreted through the lens of Gross’s Emotional Regulation Theory, it reveals a nuanced dynamic. According to the theory, effective emotional regulation involves a sequence of processes, including emotional awareness, evaluation, and regulation strategies. If students possess heightened emotional perception (a component of EI) but lack equally developed regulatory strategies, they may become more susceptible to using the internet as an emotional coping tool in stressful academic or social contexts.

In high-stress, resource-limited environments such as nursing education, emotionally aware individuals may seek emotional relief or connection through digital platforms, which can unintentionally evolve into maladaptive patterns resembling IA. This reflects an emotion-focused coping strategy, a central concept in emotional regulation theory where the regulation mechanism is present but not optimally adaptive.

Supporting this interpretation, previous studies have shown that maladaptive internet use is more common among individuals exhibiting low self-esteem, emotional instability, and impulsivity, which may result from inadequate emotion regulation skills [47, 48]. Conversely, students with higher EI levels often demonstrate better impulse control and greater ability to manage stress [49, 50], thus reducing the risk of pathological internet use.

The current study’s findings suggest that EI alone may not be uniformly protective unless all subcomponents, especially emotional regulation, are well developed. Furthermore, in the context of nursing education, emotionally intelligent students may engage more frequently with digital platforms (e.g., medical apps, online resources) as tools for academic success and social support, which may inflate IA scores while still serving adaptive purposes. This highlights the need to distinguish between functional and dysfunctional internet use, as well as the importance of contextual and coping mechanisms in shaping outcomes both of which are central to Gross’s theoretical framework.

While EI equips students with the ability to recognize and manage emotions, these same abilities may also heighten awareness of stress, academic pressures, or interpersonal difficulties. When adaptive regulation strategies (e.g., reappraisal) are insufficient, students may resort to alternative coping strategies such as excessive internet use, which functions as an immediate but maladaptive form of regulation. This perspective aligns with recent evidence suggesting that high EI can, under certain conditions, facilitate overreliance on avoidance-based coping behaviors [51]. Thus, integrating Gross’s theoretical mechanisms clarifies why EI may simultaneously promote psychological well-being while also being linked to greater IA in some students.

Thus, the integration of Emotional Regulation Theory into the analysis helps explain not only the complexity of the EI–IA relationship but also the role of coping strategies, digital engagement, and academic context in influencing student behavior. These insights support targeted interventions that promote balanced EI development, particularly enhancing emotion regulation, to buffer the potential negative effects of internet overuse.

Unlike findings in other studies, where higher EI is often associated with better self-regulation and reduced addictive behaviors, this result suggests that emotionally intelligent individuals in this setting may rely on the internet as a coping mechanism or a supportive tool, leading to increased use that could be classified as addictive. These differences indicate the importance of considering contextual and cultural factors when explaining the relationship between EI and internet use. While the current study confirms the direct relation between EI and PWB, which also agree with several studies such as Cai et al., [52] and Gioia et al., [53] who stated that nursing students who have EI can promote their PWB.

While the correlations between EI, IA and PWB in this study were found to be statistically significant, the effect sizes were small by Cohen’s (1988) standards, suggesting limited practical significance [54]. Specifically, the correlation coefficients observed fall within the small to low-moderate range, indicating limited practical significance. These findings suggest that while the variables are related, the strength of these relationships is not strong enough to imply predictive or causal inferences. Several factors may account for this; Although standardized tools were used, self-report questionnaires may introduce bias or inaccuracy due to social desirability, self-perception errors, or response fatigue.

Also, the study sample consisted solely of nursing students from a single institution, which may limit generalizability. Academic and clinical demands unique to this group may have moderated or confounded the observed relationships. EI, IA, and PWB are multifaceted constructs influenced by numerous individual, social, and environmental variables that may not have been fully captured in the present design.

Thus, we caution against over-interpreting the strength of these associations and recommend that future research explore these relationships using longitudinal designs, multi-method assessments, and more diverse samples to better understand the underlying mechanisms and practical relevance.

### Strengths and limitations of the study

Despite the strengths of this study, the use of validated tools and a relatively large sample size. several limitations should be acknowledged. First, the cross-sectional design limits the ability to establish causal relationships among emotional intelligence, internet addiction, and psychological well-being. For example, it remains unclear whether low emotional intelligence contributes to increased internet use, or whether excessive internet use may impair emotional functioning over time. Future research should adopt longitudinal or experimental designs to better examine causal pathways and temporal relationships between these variables.

Second, the reliance on self-reported data may have introduced response biases, such as social desirability or recall bias, potentially affecting the accuracy of the findings. This may have led participants to overestimate socially valued traits such as emotional intelligence, or to underreport behaviors perceived as negative, such as problematic internet use. These biases may have differential effects across variables: EI assessments, for instance, may be more susceptible to self-enhancement bias, while IA may be influenced by denial or lack of insight into addictive behaviors. Future studies could benefit from incorporating multi-method approaches, such as behavioral measures or peer assessments, to triangulate findings and reduce bias.

Third, the study was conducted at a single educational institution, which may limit the generalizability of the results to other populations of nursing students. Additionally, although the correlation between emotional intelligence and internet addiction was statistically significant, it was weak in strength, suggesting that other unmeasured factors may also influence this relationship. Finally, the sample was slightly skewed toward rural residents and females; the uneven distribution across academic years (e.g., smaller numbers in the 3rd year compared to the 4th year) may have influenced the robustness of between-year comparisons, which may have introduced bias, as differences in digital access and usage patterns across rural-urban settings and between genders could have affected the results. Therefore, caution is advised when generalizing the findings to more diverse populations.

Although the TMMS was used to assess trait meta-mood, the present study relied on the overall EI score and did not analyze the Attention, Clarity, and Repair subscales separately. This limits the ability to determine which specific facets of emotional intelligence drive the observed associations. Future research should examine these subscales individually to provide a more nuanced understanding of EI’s role in internet addiction and psychological well-being.

## Conclusion

The present study concluded that nursing students in the current study demonstrated a high level of EI, moderate level of IA and generally moderate to high well-being range. A statistically significant relationship was observed among the three variables: emotional intelligence was statistically positively correlated with IA and PWB with weak relationship indicating limited practical impact. Additionally, a negative correlation was found between IA and PWB, suggesting that excessive internet use may adversely impact on students’ mental health. The findings indicate that emotional intelligence shows a modest association with internet addiction and psychological well-being. Although statistically significant, these effects were small in magnitude. Therefore, while EI-focused interventions in nursing education may be beneficial, their impact should not be overstated, and further research is needed to confirm and expand these findings. The study reinforces existing evidence that internet addiction is negatively associated with psychological well-being. This highlights the importance of addressing problematic internet use among nursing students to protect and promote their mental health.

### Recommendations

Based on the findings of the present study, preliminary evidence suggests that emotional intelligence and internet addiction may be important factors influencing the psychological well-being of nursing students. However, before drawing firm conclusions or recommending broad interventions, it is essential to replicate this research across diverse educational and cultural settings to confirm the observed relationships. If future studies support these findings, nursing education programs might consider developing targeted strategies to help students manage internet use and strengthen emotional intelligence. Potential interventions could include emotional intelligence training modules, peer support initiatives, counseling services, and awareness campaigns promoting healthy digital habits. Such approaches, if validated through further research, may contribute to enhancing nursing students’ mental health and emotional readiness for clinical practice. Additional studies are also needed to explore the underlying mechanisms linking emotional intelligence, internet addiction, and psychological well-being in order to inform more tailored and evidence-based interventions.

## Supplementary Information

Below is the link to the electronic supplementary material.

Supplementary Material 1

## Data Availability

The datasets generated during and/or analyzed during the current study are available from the corresponding author on reasonable request.
