# Supplementary material for: The relationship between emotional intelligence, internet addiction, and psychological well-being among university nursing students
Source: BMC Nurs. 2025 Oct 28;24:1341. doi: 10.1186/s12912-025-03957-2 (PMC12570817; doi:10.1186/s12912-025-03957-2)
Supplement: Supplementary file 1 — Supplementary Material 1 [file 12912_2025_3957_MOESM1_ESM.doc]

**English version**

**Internet addiction test (20-items):**

**1. Do you feel that you stay online longer than you intend?**

**(0 = Never; 1 =Seldom; 2 = Occasionally; 3 = Frequently; 4 = very often, 5 = always)**

**2. Do you neglect household chores to spend more time online?**

**(0 = Never; 1 =Seldom; 2 = Occasionally; 3 = Frequently; 4 = very often, 5 = always)**

**3. Do you prefer the excitement of the Internet to intimacy with your partner?**

**(0 = Never; 1 =Seldom; 2 = Occasionally; 3 = Frequently; 4 = very often, 5 = always)**

**4. Do you form new relationships with fellow online users?**

**(0 = Never; 1 =Seldom; 2 = Occasionally; 3 = Frequently; 4 = very often, 5 = always)**

**5. Do others in your life complain to you about the amount of time you spend online?**

**(0 = Never; 1 =Seldom; 2 = Occasionally; 3 = Frequently; 4 = very often, 5 = always)**

**6. Does your work suffer because of the amount of time you spend online?**

**(0 = Never; 1 =Seldom; 2 = Occasionally; 3 = Frequently; 4 = very often, 5 = always)**

**7. Do you check your email before something else that you need to do?**

**(0 = Never; 1 =Seldom; 2 = Occasionally; 3 = Frequently; 4 = very often, 5 = always)**

**8. Does your job performance or productivity suffer because of the Internet?**

**(0 = Never; 1 =Seldom; 2 = Occasionally; 3 = Frequently; 4 = very often, 5 = always)**

**9. Do you become defensive or secretive when someone asks what you do online?**

**(0 = Never; 1 =Seldom; 2 = Occasionally; 3 = Frequently; 4 = very often, 5 = always)**

**10. Do you block disturbing thoughts about your life with soothing thoughts of the Internet?**

**(0 = Never; 1 =Seldom; 2 = Occasionally; 3 = Frequently; 4 = very often, 5 = always)**

**11. Do you find yourself anticipating when you go online again?**

**(0 = Never; 1 =Seldom; 2 = Occasionally; 3 = Frequently; 4 = very often, 5 = always)**

**12. Do you feel that life without the Internet would be boring, empty, and joyless?**

**(0 = Never; 1 =Seldom; 2 = Occasionally; 3 = Frequently; 4 = very often, 5 = always)**

**13. Do you snap, yell, or act annoyed if someone bothers you while you are online?**

**(0 = Never; 1 =Seldom; 2 = Occasionally; 3 = Frequently; 4 = very often, 5 = always)**

**14. Do you lose sleep due to late night log-ins?**

**(0 = Never; 1 =Seldom; 2 = Occasionally; 3 = Frequently; 4 = very often, 5 = always)**

**15. Do you feel preoccupied with the Internet when offline or fantasize about being online?**

**(0 = Never; 1 =Seldom; 2 = Occasionally; 3 = Frequently; 4 = very often, 5 = always)**

**16. Do you find yourself saying “just a few more minutes” when online?**

**(0 = Never; 1 =Seldom; 2 = Occasionally; 3 = Frequently; 4 = very often, 5 = always)**

**17. Do you try to cut down the amount of time you spend online and fail?**

**(0 = Never; 1 =Seldom; 2 = Occasionally; 3 = Frequently; 4 = very often, 5 = always)**

**18. Do you try to hide how long you’ve been online?**

**(0 = Never; 1 =Seldom; 2 = Occasionally; 3 = Frequently; 4 = very often, 5 = always)**

**19. Do you choose to spend more time online over going out with others?(0 = Never; 1 =Seldom; 2 = Occasionally; 3 = Frequently; 4 = very often, 5 = always)**

**20. Do you feel depressed, moody, or nervous when you are offline, which goes away once you are back online?**

**(0 = Never; 1 =Seldom; 2 = Occasionally; 3 = Frequently; 4 = very often, 5 = always**

**Psychological Wellbeing (18 items):**

**Instructions:** Circle one response below each statement to indicate how much you agree or disagree.

1. “I like most parts of my personality.”

| Strongly agree | Somewhat agree | A little agree | Neither agree nor disagree | A little disagree | Somewhat disagree | Strongly disagree |
| --- | --- | --- | --- | --- | --- | --- |
|  |  |  |  |  |  |  |

2. “When I look at the story of my life, I am pleased with how things have turned out so

far.”

| Strongly agree | Somewhat agree | A little agree | Neither agree nor disagree | A little disagree | Somewhat disagree | Strongly disagree |
| --- | --- | --- | --- | --- | --- | --- |
|  |  |  |  |  |  |  |

3. “Some people wander aimlessly through life, but I am not one of them.”

| Strongly agree | Somewhat agree | A little agree | Neither agree nor disagree | A little disagree | Somewhat disagree | Strongly disagree |
| --- | --- | --- | --- | --- | --- | --- |
|  |  |  |  |  |  |  |

4. “The demands of everyday life often get me down.”

| Strongly agree | Somewhat agree | A little agree | Neither agree nor disagree | A little disagree | Somewhat disagree | Strongly disagree |
| --- | --- | --- | --- | --- | --- | --- |
|  |  |  |  |  |  |  |

5. “In many ways I feel disappointed about my achievements in life.”

| Strongly agree | Somewhat agree | A little agree | Neither agree nor disagree | A little disagree | Somewhat disagree | Strongly disagree |
| --- | --- | --- | --- | --- | --- | --- |
|  |  |  |  |  |  |  |

6. “Maintaining close relationships has been difficult and frustrating for me.”

| Strongly agree | Somewhat agree | A little agree | Neither agree nor disagree | A little disagree | Somewhat disagree | Strongly disagree |
| --- | --- | --- | --- | --- | --- | --- |
|  |  |  |  |  |  |  |

7. “I live life one day at a time and don't really think about the future.”

| Strongly agree | Somewhat agree | A little agree | Neither agree nor disagree | A little disagree | Somewhat disagree | Strongly disagree |
| --- | --- | --- | --- | --- | --- | --- |
|  |  |  |  |  |  |  |

8. “In general, I feel I am in charge of the situation in which I live.”

| Strongly agree | Somewhat agree | A little agree | Neither agree nor disagree | A little disagree | Somewhat disagree | Strongly disagree |
| --- | --- | --- | --- | --- | --- | --- |
|  |  |  |  |  |  |  |

9. “I am good at managing the responsibilities of daily life.”

| Strongly agree | Somewhat agree | A little agree | Neither agree nor disagree | A little disagree | Somewhat disagree | Strongly disagree |
| --- | --- | --- | --- | --- | --- | --- |
|  |  |  |  |  |  |  |

10. “I sometimes feel as if I've done all there is to do in life.”

| Strongly agree | Somewhat agree | A little agree | Neither agree nor disagree | A little disagree | Somewhat disagree | Strongly disagree |
| --- | --- | --- | --- | --- | --- | --- |
|  |  |  |  |  |  |  |

11. “For me, life has been a continuous process of learning, changing, and growth.”

| Strongly agree | Somewhat agree | A little agree | Neither agree nor disagree | A little disagree | Somewhat disagree | Strongly disagree |
| --- | --- | --- | --- | --- | --- | --- |
|  |  |  |  |  |  |  |

12. “I think it is important to have new experiences that challenge how I think about myself and the world.”

| Strongly agree | Somewhat agree | A little agree | Neither agree nor disagree | A little disagree | Somewhat disagree | Strongly disagree |
| --- | --- | --- | --- | --- | --- | --- |
|  |  |  |  |  |  |  |

13. “People would describe me as a giving person, willing to share my time with others.”

| Strongly agree | Somewhat agree | A little agree | Neither agree nor disagree | A little disagree | Somewhat disagree | Strongly disagree |
| --- | --- | --- | --- | --- | --- | --- |
|  |  |  |  |  |  |  |

14. “I gave up trying to make big improvements or changes in my life a long time ago”

| Strongly agree | Somewhat agree | A little agree | Neither agree nor disagree | A little disagree | Somewhat disagree | Strongly disagree |
| --- | --- | --- | --- | --- | --- | --- |
|  |  |  |  |  |  |  |

15. “I tend to be influenced by people with strong opinions”

| Strongly agree | Somewhat agree | A little agree | Neither agree nor disagree | A little disagree | Somewhat disagree | Strongly disagree |
| --- | --- | --- | --- | --- | --- | --- |
|  |  |  |  |  |  |  |

16. “I have not experienced many warm and trusting relationships with others.”

| Strongly agree | Somewhat agree | A little agree | Neither agree nor disagree | A little disagree | Somewhat disagree | Strongly disagree |
| --- | --- | --- | --- | --- | --- | --- |
|  |  |  |  |  |  |  |

17. “I have confidence in my own opinions, even if they are different from the way most

other people think.”

| Strongly agree | Somewhat agree | A little agree | Neither agree nor disagree | A little disagree | Somewhat disagree | Strongly disagree |
| --- | --- | --- | --- | --- | --- | --- |
|  |  |  |  |  |  |  |

18. “I judge myself by what I think is important, not by the values of what others think is

important.”

| Strongly agree | Somewhat agree | A little agree | Neither agree nor disagree | A little disagree | Somewhat disagree | Strongly disagree |
| --- | --- | --- | --- | --- | --- | --- |
|  |  |  |  |  |  |  |

**Trait meta-mood scale**

|  | **1= Never** | **2= Rarely** | **Sometimes 3=** | **Often 4=** | **5=Always** |
| --- | --- | --- | --- | --- | --- |
| **Emotional attention**  1-I pay more awareness to feelings. |  |  |  |  |  |
| 2- I usually care a lot of how I feel. |  |  |  |  |  |
| 3- I usually waste time thinking my sentiments |  |  |  |  |  |
| 4- I think deserve to have attention to my feelings and mood |  |  |  |  |  |
| 5- My feelings affect my thoughts. |  |  |  |  |  |
| 6-I continue thinking of my state of mind |  |  |  |  |  |
| 7- I often think touching my feelings |  |  |  |  |  |
| 8- I pay attention to how I feel. |  |  |  |  |  |
| **Feelings Clarity**  9- I am clear on my feelings. |  |  |  |  |  |
| 10- I can often determine my feelings |  |  |  |  |  |
| 11- My feelings and actions are appropriate |  |  |  |  |  |
| 12- My specific feelings are correct towards people |  |  |  |  |  |
| 13- I often notice my feelings in varying situations |  |  |  |  |  |
| 14- I can describe the feelings rounding in my mind |  |  |  |  |  |
| 15- Sometimes I can know what my feelings are |  |  |  |  |  |
| 16- I can understand my emotions |  |  |  |  |  |
| **Mood repair**  17- I have an optimistic thought, no  matter how anxious I feel |  |  |  |  |  |
| 18-Even if I feel wrong, I try to think like beautiful things |  |  |  |  |  |
| 19- When I feel depressed, I think of all life pleasures |  |  |  |  |  |
| 20-I try to have positive opinions even if I feel bad |  |  |  |  |  |
| 21-I am looking for calming me down if everything gets complicated |  |  |  |  |  |
| 22-I was afraid of becoming a good mood |  |  |  |  |  |
| 23-I have more strength when I feel happy |  |  |  |  |  |
| 24- When I am angry, I try to change my mood in the front of others |  |  |  |  |  |
